# Motor priming induces changes in resting state dynamics in cerebellum of writer’s cramp dystonia: insights for clinical therapies

**DOI:** 10.3389/dyst.2025.14533

**Published:** 2025-09-09

**Authors:** Patrick J. Mulcahey, Michael Fei, James T. Voyvodic, Michael W. Lutz, Noreen Bukhari-Parlakturk

**Affiliations:** 1 Medical Scientist Training Program, Duke University School of Medicine, Durham, NC, United States,; 2 Department of Neurology, Duke University School of Medicine, Durham, NC, United States,; 3 Department of Radiology, Duke University School of Medicine, Durham, NC, United States,; 4 Brain Imaging and Analysis Center, Duke University School of Medicine, Durham, NC, United States,; 5 Duke Institute for Brain Sciences, Duke University, Durham, NC, United States

**Keywords:** writer’s cramp, functional network, superior parietal cortex, cerebellum, amplitude of low frequency fluctuation

## Abstract

**Background:**

Writer’s cramp (WC) is an adult-onset focal dystonia that impairs hand and arm movements during writing tasks. Previous studies have identified abnormal resting state connectivity between the basal ganglia and cerebellum in WC. However, the role of brain state in modulating these dynamics remains poorly understood, limiting advances in clinical therapies.

**Objective:**

This study investigated how a motor priming paradigm affects resting state dynamics in functional networks implicated in WC dystonia.

**Methods:**

Fourteen WC participants and 20 HV underwent functional MRI (fMRI) during two resting brain states: naïve rest and rest following a motor priming paradigm (non-naïve rest). Group Independent Component Analysis was applied to isolate independent spatial components called functional networks (FNs) known to play a role in dystonia including the cerebellar, basal ganglia, sensorimotor, and superior parietal networks. The default mode network served as a control. To compare resting state brain dynamics between WC and HV, amplitude of low frequency fluctuations (ALFF) were extracted for each FN and resting state. Frequency- and state-dependent differences in ALFF were statistically assessed using general linear modeling.

**Results:**

At 0.02 Hz, ALFF differences in the cerebellar network were influenced by both group identity and rest condition, with WC exhibiting decreased values during non-naïve rest, opposite to the pattern observed in HV. In contrast, at 0.10 Hz, WC showed increased ALFF values in the superior parietal network compared with HV, and this difference was independent of the motor priming paradigm.

**Conclusion:**

Brain state significantly influences resting state dynamics in WC dystonia, with the cerebellar and superior parietal networks exhibiting distinct state- and frequency-dependent engagement. These findings highlight the importance of integrating motor priming paradigms with neuromodulation therapies to selectively engage key brain networks implicated in WC dystonia and potentially improve therapeutic outcomes.

## Introduction

Focal hand dystonia (FHD) is an adult-onset dystonia characterized by involuntary muscle contractions that result in abnormal, repetitive, and often painful postures of the fingers, hand and arm [1]. Writer’s cramp (WC) is a task-specific form of focal hand dystonia with patients manifesting dystonic symptoms primarily during writing task [2]. Despite its debilitating impact, current therapies such as medications and neurotoxin injections offer limited benefit with poor side effect profiles [3]. To develop more effective treatments for dystonia, there is a critical need for neuroimaging studies to identify pathological brain regions that could serve as targets for therapeutic intervention.

Resting state functional MRI (rest fMRI) has emerged as a powerful tool for investigating the neural mechanisms of dystonia. Prior studies in focal adult dystonias identified abnormalities in functional connectivity during naïve rest. Specifically, patients with focal adult dystonias exhibit decreased connectivity between the sensorimotor cortex and the superior parietal cortex compared to healthy volunteers (HV) [4]. Dresel and colleagues also reported decreased connectivity between the sensorimotor cortex and superior parietal cortex, and increased connectivity between the sensorimotor and cerebellum [5]. More recently, Mantel and colleagues used a data driven approach called independent components analysis (ICA) to observe reduced connectivity between the functional networks (FNs) of basal ganglia and cerebellum in WC compared to HV [6]. Collectively, these rest-fMRI studies identified key abnormalities in the brain regions sensorimotor cortex, and superior parietal cortex, and FNs of basal ganglia, and cerebellum during naïve rest in WC dystonia.

Although prior studies have identified brain abnormalities during naïve rest, the resting-state brain signal following a motor task—referred to as non-naïve rest—remains largely unexplored in dystonia. The non-naïve rest state provides unique insights into how behavioral priming paradigms influence resting state brain function [7]. For example, a rest fMRI study in healthy adults demonstrated increased connectivity between the left and right motor cortices following a behavioral priming paradigm compared to naïve rest [8]. Behavior priming paradigms are particularly relevant in task-specific dystonias like WC, where clinical symptoms are triggered by specific motor tasks and may reflect state-dependent changes in brain dynamics that are not evident during naïve rest. Our group also recently performed a clinical trial applying transcranial magnetic stimulation (TMS) in WC patients. To engage the relevant brain networks, we aimed to deliver TMS while WC patients were engaged in the writing task. Since TMS cannot be accurately delivered during a motor task, TMS delivery was interleaved with the writing task. The writing task, therefore, served as a motor priming paradigm and TMS was ultimately delivered during a non-naïve resting brain state. Importantly, delivery of TMS during non-naïve rest state led to improvement in dysfluent writing behavior in WC [9]. Both prior observational studies and our recent clinical trial highlight the importance of understanding the brain dynamics in non-naïve resting brain state and its role in future clinical interventions.

Functional connectivity is a common approach to study resting-state brain function but is limited in its ability to resolve abnormalities within functional networks. While functional connectivity analyzes BOLD signal of highly correlated brain regions within or between functional networks, the amplitude of low-frequency fluctuations (ALFF) provides a complementary approach to study the strength of resting state brain signal within a functional network [10–12]. Biologically, ALFF values represent spontaneous neural activity of a brain network with higher values representing greater neural activity in a functional network [13]. Changes in ALFF values in key brain regions were demonstrated to be an early predictor of brain pathology in neonatal brain disorder and Alzheimer’s disease [14, 15]. ALFF values, therefore, can be used to gain mechanistic insight on the neural basis of a behavior priming paradigm and identify key functional networks engaged by a motor priming paradigm which in turn can serve as targets for future clinical interventions.

In this study, we examined how a motor priming paradigm influenced resting state brain dynamics in a cohort WC dystonia and healthy participants. We compared two distinct brain states: naïve rest (baseline) and non-naïve rest (following a motor priming paradigm). We hypothesized that the five FNs of interest will demonstrate differences in ALFF values due to group identity and rest condition. Rest-fMRI data from both rest-states were analyzed using group independent component analysis (ICA). Group ICA is a data-driven method to identify independent spatial components called functional networks (FNs) [16]. FNs for the cerebellum, basal ganglia, sensorimotor cortex, superior parietal cortex and as a control, default mode network were selected for spectral power analysis. For each FN and group (HV and WC), ALFF values between naïve rest and non-naïve rest were compared.

## Methods

### Participants

The study was approved by the Duke Health Institutional Review Board (IRB: 0094131) and performed in accordance with the Declaration of Helsinki. All enrolled participants gave written informed consent. Inclusion criteria were isolated right-hand dystonia during the writing task diagnosed by a Movement Disorder Specialist, more than 3 months from the last botulinum toxin injection, and more than 1 month from trihexyphenidyl medication. Exclusion criteria were any contraindications to receiving MRI. Aged-matched, right-hand dominant HVs without structural brain disorders or psychiatric illnesses were also recruited for the study. A total of 39 participants consented to the study (22 HV and 17 WC).

### Rest-fMRI study design

All participants completed two ten-minute rest fMRI scans. During the first rest fMRI (naïve rest), participants stared at a fixation cross while keeping their hands at rest. CIGAL software was used to present the fixation cross [17]. After completion of the first rest fMRI, participants performed three sequential motor tasks of decreasing complexity: writing, four-button sequence typing and finger flexion-extension over 22 min with their right hand (Figure 1). This hierarchical design was chosen to capture the spectrum of dystonia severity in patients. Patients with simple writer’s cramp only manifest dystonic symptoms during writing while patients with complex writer’s cramp manifest dystonic symptoms during both writing, and other complex motor tasks such sequence typing. Patients with severe dystonia display dystonic symptoms even during simple motor tasks such as finger flexion-extension. Therefore, three motor tasks ranging from complex motor skill of writing to complex motor movement of (sequence typing) and simple motor task (flexion-extension) were performed by participants to differentially engage brain networks important for hand movement. This paradigm allows us to evaluate how resting state brain dynamics was influenced by recent motor engagement, providing insights into state-dependent and frequency-dependent changes in resting state brain function. The performance of all three tasks by participants was monitored using an MRI compatible tablet and button box, the output of which was visible to the study team in real time outside the MRI scanner. At the end of each MRI, the study team member documented that the study participant performed all three motor tasks. The three motor tasks were followed by a second rest fMRI scan (non-naïve rest) during which participants stared again at a fixation cross while keeping their hands at rest (Figure 1).

### Image acquisition

Structural and functional MRI were acquired from all participants using a 3-Tesla GE scanner equipped with an 8-channel head coil. Structural T1-weighted images were acquired using a BRAVO imaging sequence with the following parameters: 256 × 256 matrix, repetition time (TR) = 7.316 s; echo time (TE) = 3.036 s; field of view (FOV) = 25.6 mm^2^, bandwidth 41.67 Hz/Pixel, 1 mm slice thickness. Functional echo-planar images were acquired using the following parameters: voxel size = 3.5 × 3.5 × 4.0 mm^3^, TR = 2 s, TE = 30 ms, flip angle = 90°, FOV = 22 cm, bandwidth = 250 Hz/Pixel, 37 interleaved slices, total run time: 10 min.

### Image preprocessing

Before starting with the MRI analysis, the T1, and rest fMRI images were structured according to the Brain Imaging Data Structure (BIDS) specifications [18]. Preprocessing of rest fMRI data was performed using the fMRIPrep toolbox 20.2.0 with the default processing steps utilizing the software packages FSL, FreeSurfer, ANTs, and AFNI [19]. For further details on each preprocessing step in fMRIprep, please refer to the online documentation under.^1^ Briefly, a reference volume and its skullstripped version were first generated. The BOLD reference image was then co-registered to the T_1_-weighted anatomical reference. Head-motion parameters with respect to the BOLD reference (transformation matrices, and six corresponding rotation and translation parameters) were estimated before any spatiotemporal filtering. The BOLD runs were then slice-time corrected and finally resampled into MNI152NLin2009cAsym standard space with a voxel size of 3.5 mm × 3.5 mm × 4 mm^3^.

Several confounding time-series were calculated during preprocessing: cerebrospinal fluid, white matter, framewise displacement (FD), and motion outliers were extracted for whole-brain masks, which were later used as nuisance regressors. After data collection, participants with excessive head movements defined by mean frame-wise displacement >0.3 mm were excluded from the study. This threshold resulted in five participants, two HV and three WC, being excluded from the data analysis.

### Generation of independent functional brain networks

Group spatial independent component analysis (ICA) on the rest fMRI of each WC and HV was performed using GIFT v3.0 Toolbox on Matlab R2019b [16]. ICA estimates spatially maximally independent sources from the linearly mixed signals contained in a spatiotemporal fMRI dataset, providing spatial maps of temporally coherent brain regions (functional networks) [16]. The calculated spatially independent components represent either meaningful (i.e., intrinsic connectivity networks (ICNs)) or spurious (e.g., noise) information. As a first step, the number of independent components in the dataset was established. The infomax algorithm in GIFT toolbox estimated 40 independent components. GIFT toolbox was then used to generate 40 independent components. To distinguish ICNs from spurious components, the spatial maps of each component was correlated with publicly available maps of ICNs identified in a meta-analysis of fMRI studies performed by Laird and colleagues using multiple regression [20–23]. The non-noise independent component with the best fit (highest coefficient of determination) was selected. Independent components representing noise were identified by standardized visual inspection of their spatial (activation pattern and tissue overlap with grey matter (GM)) and temporal characteristics (e.g., presence of saw tooth and high frequency patterns or spikes) as previously described [24]. Of the 40 ICNs generated, 30 had spatial distributions that visually were consistent with known ICNs. Of the 30 identified ICNs, the ICNs for cerebellum (CBL, ~Laird’s ICN14), basal ganglia-thalamus (BG, Laird’s ICN3), superior parietal cortices (SPC, Laird’s ICN 9), and sensorimotor cortex (SMC, Laird’s ICN 8) were selected for further analysis based on prior studies implicating their role in adult focal dystonias [6]. The default mode network (DMN, Laird’s ICN 13) was selected as a positive control since two prior studies have reported no significant differences in network signal between healthy and focal dystonias [25, 26] (Figure 2). The ICASSO toolbox was then used to test the reliability of these ICNs [27]. Specifically, group ICA was repeated 30 times, the known ICNs were clustered, and their quality was quantified using the index Iq (range 0–1). The Iq index reflects the difference between intra- and extra-cluster similarity with 1 representing high intra-cluster and low extra-cluster reliability. All k ICNs had Iq >0.95, indicating high-quality, stable functional network estimations.

### Generation of time frequency plots

From the GIFT toolbox, the time courses for each subject, rest-state and FN of interest was extracted. The nuisance regressors of white matter, cerebrospinal fluid, framewise displacement, and motion outlier variables were regressed out from these time courses using a linear model. The regressed time course data was then used to estimate time frequency plots from 0.01 to 0.1 Hz for each rest fMRI, each subject and each FN of interest using the MATLAB function spectrogram(), with a window length of 100 samples and an overlap of 80 samples [6, 19, 28, 29]. Time frequency plots from one WC and one HV were presented for the five FNs of interest.

### Analysis of amplitude of low frequency fluctuations (ALFF)

The regressed time course data for each subject, FN, and rest state was also fast fourier transformed using the MATLAB function *fft*. The ALFF data was then interpolated from 0.01 to 0.1 Hz in steps of 0.01 Hz and log base transformed for each subject, FN and rest-state. The log base 10 transformed ALFF data was presented graphically and used for statistical analysis [6, 19, 28, 29].

### Statistical analysis

To evaluate for age differences between HV and WC, an unpaired t-test was performed, and equal variance was not assumed. To evaluate for sex differences, Fisher exact test was performed. To determine the frequency band to analyze for group differences in ALFF values, a Cohen’s D was calculated across all FNs and rest states. To focus on clinically meaningful differences, frequency bands that showed large group differences in ALFF values (defined as Cohen’s D > |0.8|) were selected for statistical analysis. Statistical differences in ALFF values due to group identity and rest condition were then calculated using general linear modeling. In each GLM analysis, the ALFF value of the FN was the dependent variable and the covariates were rest condition (naïve vs. non-naïve), group (HV vs. WC) and interaction term (rest condition*group). Statistical significance was defined as p-values <0.05.

## Results

### Participant clinical characteristics

Data from 20 HV to 14 WC were used for brain imaging analysis with no significant differences in mean age (t = −4.42, p = 0.330) or sex (p = 0.275, Fisher exact test) (Table 1). All subjects in the study were right-handed. WC subjects showed a mean disease duration of 20 years [standard deviation (SD) 14 years]. Technical differences in fMRI signal between the two study groups was measured using the head motion parameter of mean framewise displacement. No differences in mean framewise displacement were observed between WC and HV (naïve: t = 0.012 p = 0.527; non-naïve rest: t = 0.022 p = 0.296). Therefore, functional brain signal during rest-fMRI across the two groups during naïve and non-naïve rest was technically comparable.

### Motor priming paradigm changed resting state spectral power in cerebellum and superior parietal cortex

After group ICA extraction and removal of head motion parameters, the time courses of the FNs were used to generate time-frequency plots and visualize dynamic changes in spectral power along the frequency domain and rest states. Representative time-frequency plots for a HV and a WC participant are shown in Figure 3. These time-frequency plots demonstrate three key points. First, both HV and WC exhibit higher spectral powers in the low frequency bands. Two, WC qualitatively showed differences in their resting state spectral power compared to HV. Specifically, during naïve-rest, WC show higher spectral power in the CBL, SMC and SPC FNs compared to HV (Figure 3, naïve rest panels). Three, performance of a motor priming paradigm in HV and WC changed the pattern of resting state spectral power across all FNs (Figure 3, non-naïve rest panels).

To quantify group differences in resting state spectral power, ALFF values were calculated for each subject, rest state, and FN of interest (Figure 4). Frequency bands that showed large differences in ALFF values between HV and WC (defined as Cohen’s D > |0.8|) were selected for statistical analysis of spectral power. As a result, the frequency band of 0.02 Hz in the CBL FN and 0.10 Hz in the SPC FN met the criteria for further analysis of ALFF values.

A statistical analysis of ALFF values in CBL FN at 0.02 Hz showed no significant differences due to group identity (estimate: −0.12, SE: 0.063, p = 0.052) or rest condition (estimate = 0.04, SE 0.063, p = 0.564) but a significant interaction between group and rest condition (estimate: −0.125, SE 0.063, p = 0.0498) (Figure 5A). These findings suggest that the differences in ALFF values in the CBL were due to both group identity and rest condition with higher ALFF values observed in HV during non-naïve rest and lower ALFF values observed in WC during non-naïve rest. For the superior parietal FN at 0.10 Hz, a significant difference in ALFF values were observed due to group identity (estimate: −0.12, SE: 0.041, p = 0.0059) but not rest condition (0.022, SE 0.041, p = 0.592) or interaction between group and rest condition (estimate: 0.070, SE 0.041, p = 0.092) (Figure 5B). These findings suggest that the differences in ALFF values in the SPC were due to group identity only and irrespective of naïve or non-naïve rest.

Collectively, these findings demonstrate that a motor priming paradigm differentially affects resting state spectral power in the CBL of HV and WC participants. Group differences in the spectral power are also observed in SPC FNs but these differences are irrespective of the motor priming paradigm.

## Discussion

This study investigated how a motor priming paradigm affected resting-state brain function in writer’s cramp dystonia. Using amplitude of low-frequency fluctuations, we identified spectral power differences in the cerebellar network that depended on both performance of a motor priming paradigm and group identity. In addition, spectral power in the superior parietal cortex network was increased in writer’s cramp patients compared to healthy volunteers irrespective of performance of a motor priming paradigm. Collectively, these findings suggest that performance of a motor priming paradigm in task-specific focal hand dystonia may selectively engage deep brain networks such as the cerebellum. Non-invasive brain stimulation techniques such as TMS may, therefore, improve modulation of deep brain regions in dystonia by interleaving stimulation delivery with a motor priming paradigm.

Our primary study finding is that the cerebellar network showed differences in spectral power that depend on both performance of a motor priming paradigm and group identity. Specifically, WC subjects demonstrate decreased spectral power in the cerebellar network during non-naïve rest compared to naïve rest. In contrast, HV demonstrated increased spectral power in the cerebellar network during non-naïve rest compared to naïve rest. The motor priming paradigm, therefore, had opposite effects on the cerebellar network at rest based on group identity. The differential effect of a motor priming paradigm demonstrate that the resting cerebellar network is dysfunctional in WC dystonia and this novel brain response can be harnessed to improve clinical response to neuromodulation therapies. Indeed, in our prior TMS study, we observed significant changes in cerebellar connectivity after active TMS compared to sham TMS further supporting that alternating stimulation blocks with a motor priming paradigm may allow us to uniquely engage and modulate the deep cerebellar network. In summary, the present study finding of a differential effect of the motor priming paradigm in the cerebellum could be harnessed to improve response to neuromodulation therapies. Specifically, in WC patients undergoing TMS, performance of a motor priming paradigm interleaved with TMS delivery may allow for engagement and modulation of the deep cerebellar functional network and thereby, improve therapeutic outcomes in patients.

Another key study finding was increased spectral power in the functional network of superior parietal cortex in WC compared to healthy and this relationship was irrespective of a motor priming paradigm. Future studies are needed to better understand all the brain regions activated in the superior parietal cortex network. However, at minimum, the superior parietal cortex is known to play a critical role in manipulation of working memory [30, 31]. Specifically, the superior parietal cortex integrates somatosensory information about the hand into motor planning [30, 31]. The elevated spectral power in the functional network of the superior parietal cortex during both naïve and non-naïve rest periods may be a manifestation of the abnormal sensorimotor integration in WC and serve as an endophenotype for this brain disorder. Interestingly, in our prior study applying 10 Hz TMS in WC dystonia, we reported a strong correlation between BOLD activity at the superior parietal cortex and dysfluent writing behavior after sham-TMS and this correlation was reduced after active-TMS. Therefore, the present observational study and previous interventional study support a key role for the superior cortex network.

The frequency-specific alterations in resting-state power highlight the dynamic nature of dystonia-related brain abnormalities. In WC patients, decreased power in the cerebellar network was observed at 0.02 Hz, a frequency band linked to long-range neural communication, such as connection with the sensorimotor network [32]. This suggests that cerebellar network dysfunction in the ultra-low-frequency band may underlie the impaired connectivity with the sensorimotor network previously reported in other studies [5, 6]. In contrast, the superior parietal network exhibited increased power at 0.10 Hz, a frequency band associated with task-related neural synchronization [33]. Superior parietal network dysfunction in the higher frequency band may, therefore, contribute to the deficits in sensorimotor integration clinically observed in WC. Together, these frequency-dependent changes—observed in the cerebellar network and the superior parietal network—provide valuable insights into the complexity of dystonia-related brain abnormalities and their relationship to clinical symptoms.

The state- and frequency-dependent changes in brain power observed in this study carry significant implications for developing targeted clinical therapies for dystonia. Current neuromodulation approaches, such as transcranial magnetic stimulation and deep brain stimulation, typically target specific brain networks without accounting for the differential effect brain state may have on engaging brain networks. Our findings suggest that integrating neuromodulation therapies with motor priming paradigms could more effectively engage key brain networks implicated in task-specific dystonias, potentially enhancing therapeutic outcomes. For instance, targeting the cerebellum following a motor priming paradigm may align treatment delivery with the brain states most relevant to dystonia pathophysiology, thereby optimizing clinical results.

The application of group ICA and ALFF analysis in this study established a robust framework for examining state- and frequency-dependent changes in brain power. By isolating key functional networks and quantifying their ALFF values, we identified distinct patterns of functional network engagement in WC dystonia that might have been missed using traditional functional connectivity methods. Furthermore, the incorporation of a motor priming paradigm enabled us to investigate how recent motor activity modulates resting-state brain dynamics, offering a more comprehensive perspective on the neural mechanisms underlying task-specific dystonia.

While this study offers valuable insights into the brain mechanisms underlying WC, several limitations should be acknowledged. First, the study cohort, though consistent with prior neuroimaging studies in dystonia, is relatively small. Future studies with larger cohorts are needed to validate these findings and explore the effects of behavior priming paradigms in other types of focal adult dystonias. Second, while the motor priming paradigm of writing, typing, and flexion-extension tasks effectively revealed state-dependent brain changes on fMRI, it may not fully capture the complexity of other dystonic-inducing motor behaviors. Incorporating ecologically valid tasks, such as music playing, into fMRI protocols could provide deeper insights into the neural correlates of other task-specific dystonias. Third, the two rest fMRIs were performed in a fixed design with naïve rest performed first and non-naïve rest performed after a motor priming paradigm. This fixed design was necessary as naïve rest by definition has to be performed prior to any motor tasks. But the fixed design may have led to fatigue or boredom for the study participants and affected the non-naïve rest scans. Any effects of fatigue and boredom, however, would have been experienced across both HV and WC alike. Finally, although ALFF analysis provides critical insights on brain power, integrating complementary measures, such as functional connectivity and graph theory, in future studies with larger study cohort and longer rest-fMRI sequences could offer a more comprehensive understanding of network-level abnormalities in resting-state brain dysfunction in focal adult dystonias.

In summary, this study demonstrates that brain state significantly influences the engagement of key brain networks in writer’s cramp dystonia. The cerebellar network and superior parietal network exhibit distinct state- and frequency-dependent changes in resting-state brain dynamics. These findings highlight the importance of incorporating motor priming paradigms into the development of targeted clinical therapies for dystonia. By aligning clinical interventions with specific brain states and frequency bands, it may be possible to enhance the precision and efficacy of neuromodulation therapies, ultimately improving outcomes for individuals with writer’s cramp dystonia.

## Figures and Tables

**FIGURE 1:**
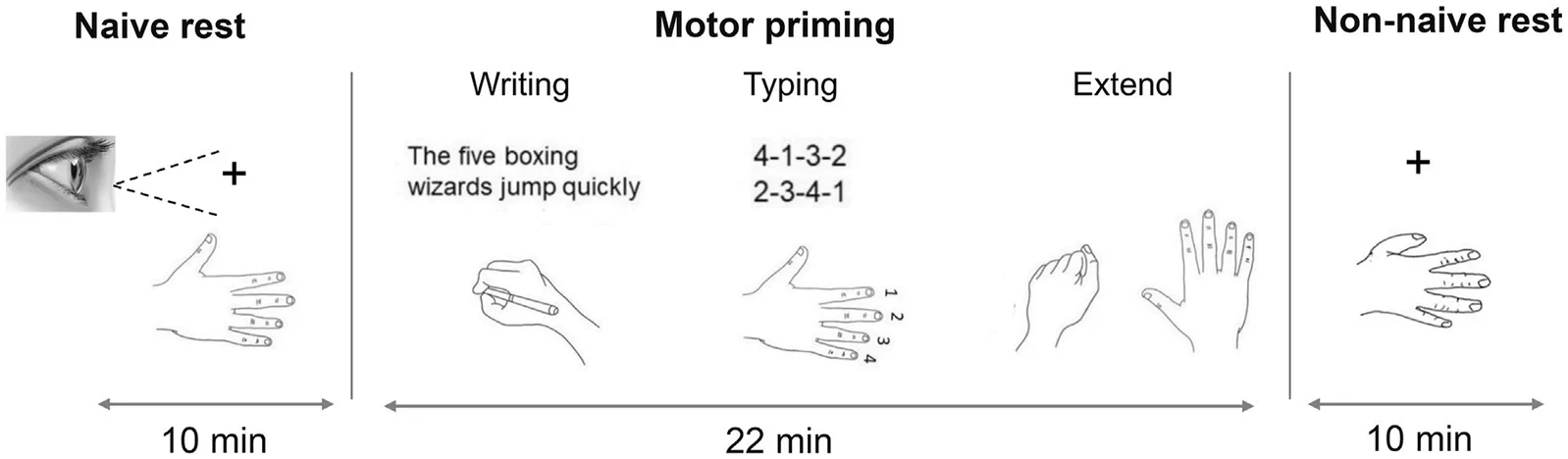
Schematic diagram of the resting state fMRIs performed before and after a motor priming paradigm. Each participant performed rest-fMRI before (naïve-rest) and after (non-naïve rest) a motor priming paradigm. During the two rest-fMRIs, participants kept their right hand at rest while staring at a fixation cross. During the motor priming paradigm, instructions were presented on a screen. *Writing:* participants copied a holoalphabetic sentence on a digital writing tablet. *Typing:* participants typed four digit sequences every 4 seconds. *Extend:* participants mimicked an image of the right hand flexing and extending.

**FIGURE 2:**
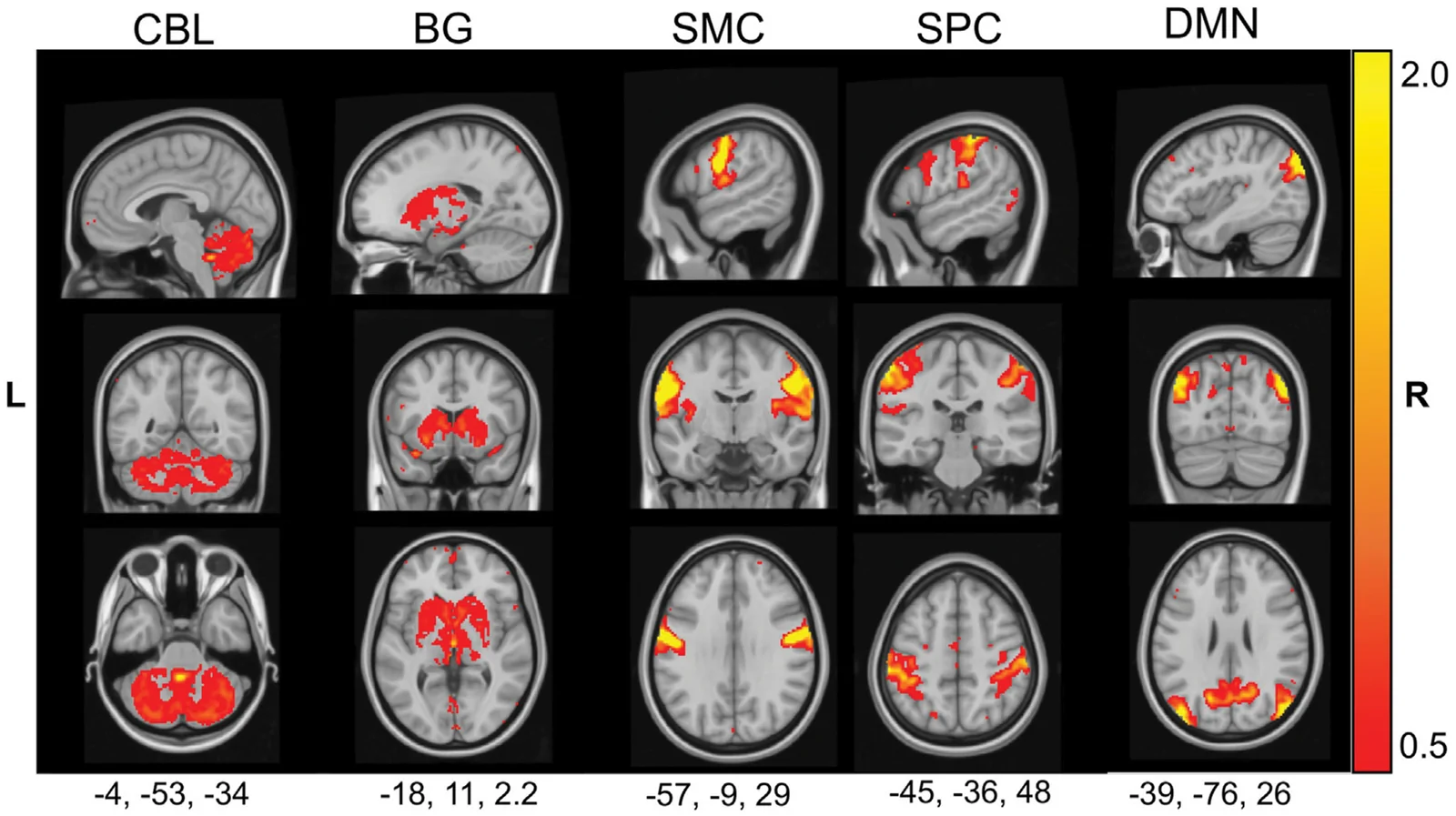
Five functional networks of interest. The activation pattern of five functional networks identified by GICA are shown in sagittal, coronal and axial planes (MNI coordinates are listed below each functional network) and according to neurological convention. The functional networks include cerebellum (CBL), basal ganglia (BG), sensorimotor cortex (SMC), superior parietal cortex (SPC), and default mode network (DMN).

**FIGURE 3:**
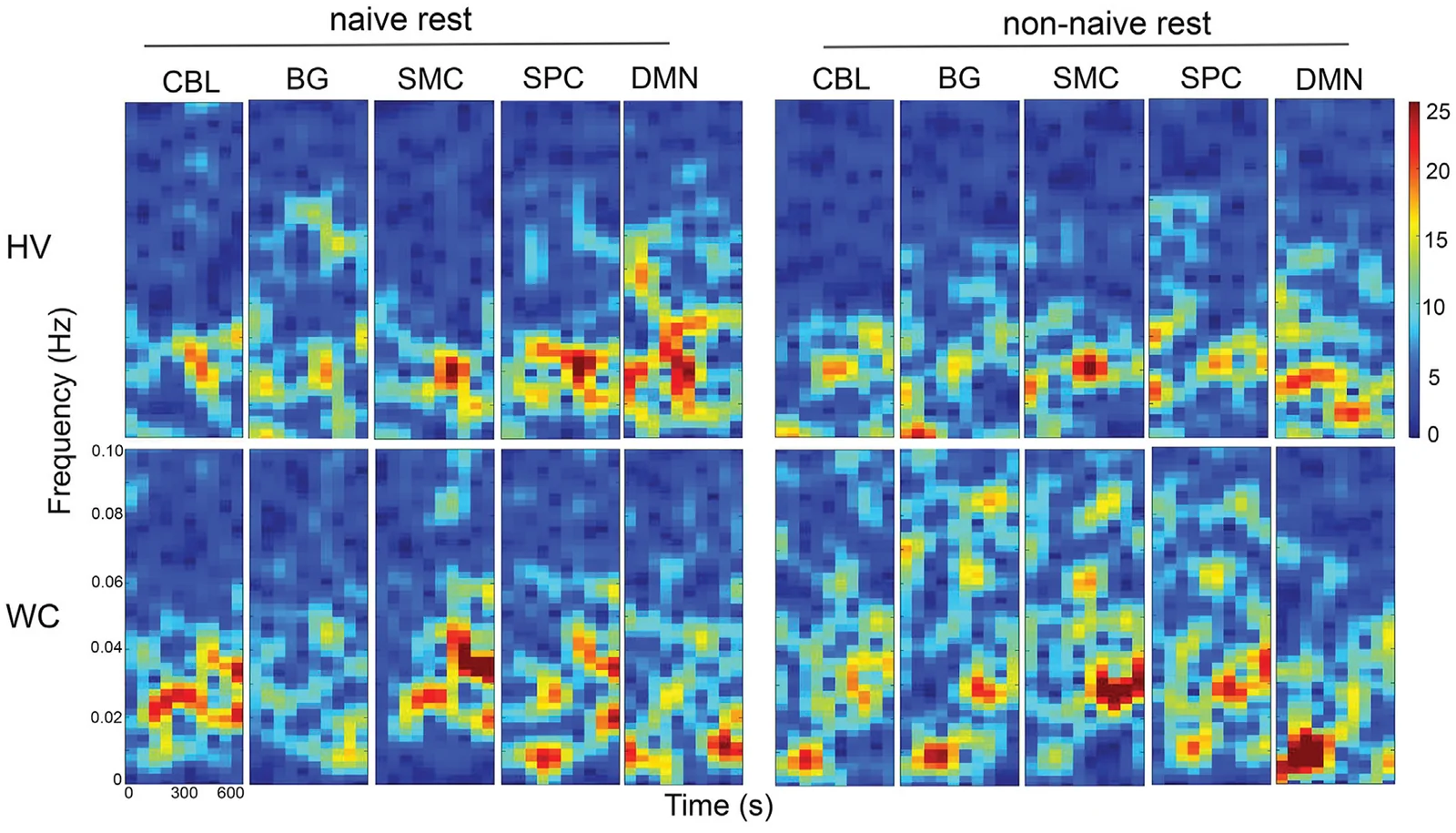
Representative time-frequency plots across five functional networks in WC and HV during two different rest states. A representative time frequency plot for the five functional networks was generated from a WC and HV participant. Each plot presents the resting state brain pattern across the frequency domain (y-axis: 0.01–0.1 Hz) and time (x-axis: 600 s) with the color scale indicating the amplitude of the frequency bands also known as the power. The time frequency plots demonstrate different patterns of brain power during naïve and non-naïve rest. These differences were observed during specific frequency bands for each FN in both HV and WC participants.

**FIGURE 4:**
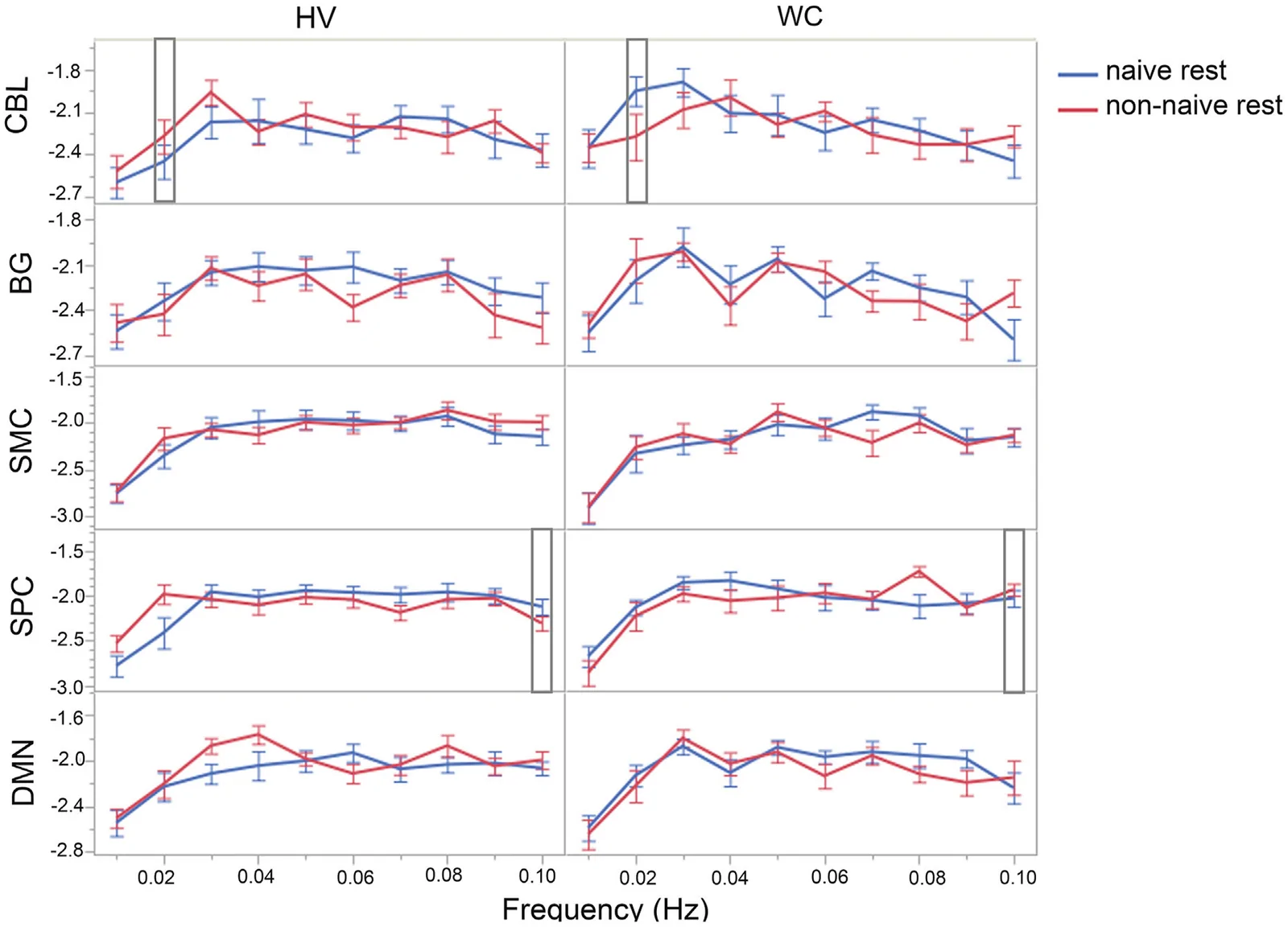
WC participants exhibit brain-state dependent changes in spectral power in the cerebellum and superior parietal cortex. Line graph shows differences in spectral power in five functional networks of interest in HV and WC during naïve rest and non-naïve rest. The spectral power across all frequency bands and functional networks were compared to determine which frequency bands show largest differences in ALFF values (Cohen’s D > 0.8) between HV and WC. The frequency band of 0.02 Hz in the cerebellum and 0.10 Hz in superior parietal cortex (indicated by rectangle bars) showed largest differences in spectral power across all frequency bands and functional networks and were selected for further analysis. Data are presented as log10(ALFF) and error bars represent standard error of the mean.

**FIGURE 5:**
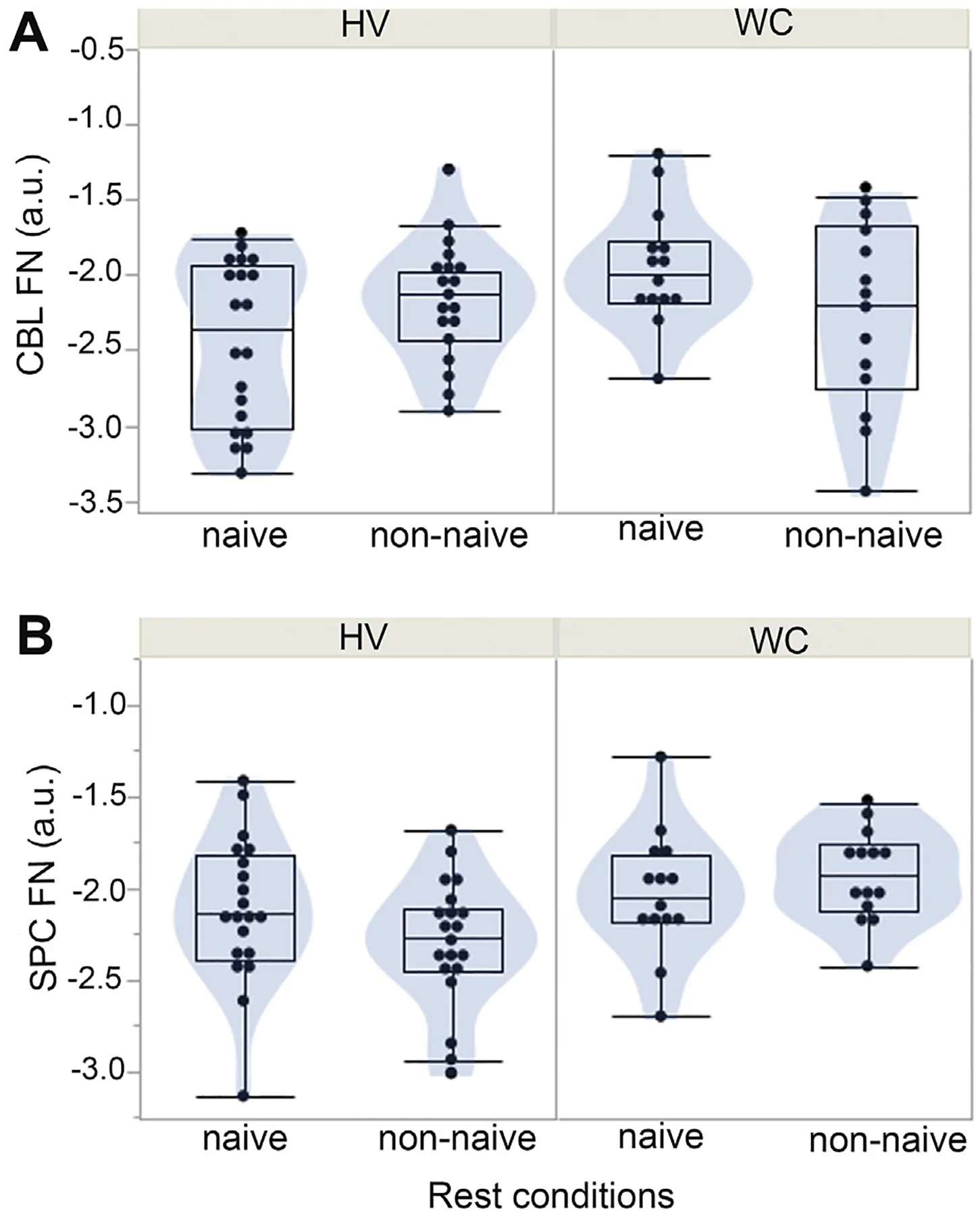
A motor priming paradigm differentially affects resting-state spectral power in the cerebellum and superior parietal cortex of WC. **(A)** Differences in spectral power in the cerebellum functional network were dependent on the group identity and rest condition with higher values observed in HV during non-naïve rest and lower values observed in WC during non-naïve rest. **(B)** For the superior parietal cortex functional network (SPC FN), WC exhibited increased spectral power compared to HV but no differences were observed due to rest condition or interaction term.

**TABLE 1:** Study Cohort and MRI motion parameters.

Demographics			
Categories	HV	WC	p-value
n	20	14	
Age (yrs)	62 [12]	57 [13]	0.330
Gender (F/M)	9/11	3/11	0.275
Disease Duration (yrs)	–	19 [14]	
MRI Framewise Displacement (mm)			
Rest sequence	HV	WC	p-value
Naïve rest	0.15 [0.05]	0.15 [0.06]	0.527
Non-naive rest	0.14 [0.05]	0.16 [0.07]	0.296

Data presented as mean [SD].

## Data Availability

The raw data supporting the conclusions of this article will be made available by the authors, without undue reservation.

## Footnotes

1
https://fmriprep.org/en/stable/
